# Dementia Care in an Aging World: Achievements, Challenges, and Future Directions

**DOI:** 10.1111/ggi.70506

**Published:** 2026-04-28

**Authors:** Hajime Takechi

**Affiliations:** ^1^ Department of Geriatrics and Cognitive Disorders Fujita Health University School of Medicine Toyoake Aichi Japan

**Keywords:** basic act on dementia, dementia care pathway, dementia‐friendly community, human rights–based approach, person‐centered care

## Abstract

As populations age worldwide, the number of people living with dementia continues to increase. Given the complex pathophysiology of dementia and its profound impact on individuals, families, and society, Japan and many other countries have developed and implemented dementia‐related policies over the past two decades. Specific initiatives include the development of dementia care pathways, early‐stage intensive support teams for community‐dwelling people with dementia, dementia cafés, dementia care teams for hospitalized physically ill people with dementia, and workforce development programs related to dementia care. Building on these efforts, opportunities for people living with dementia to express their perspectives and actively participate in society have also expanded. As a result of these cumulative initiatives, Japan enacted the Basic Act on Dementia in 2023 and is currently in the process of formulating and implementing a national dementia plan based on this legislation. This review summarizes the achievements to date in dementia care, clarifies current challenges, and discusses future directions for dementia policy and practice. These experiences from Japan, one of the world's most advanced aging societies, may provide important insights for other countries facing rapid population aging.

## 1 Introduction

With the aging of societies, the number of people living with dementia continues to increase worldwide. It was estimated that 55 million people were living with dementia globally in 2019, and this number is projected to reach 139 million by 2050 [1]. In Japan, epidemiological studies have estimated that 12.3% of older adults, approximately 4.43 million people, are living with dementia, making it a highly prevalent condition [2]. Dementia is typically a slowly progressive brain disorder that gradually leads to impairment in multiple aspects of daily functioning [3, 4]. In addition to cognitive decline, behavioral and psychological symptoms, including agitation and aggression, often emerge as a result of neurodegenerative changes and interactions with family members and the surrounding environment [3, 4]. As these symptoms progress, people with dementia often find it increasingly difficult to live independently and require support from family members, healthcare professionals, and social care providers [3, 4].

For the general public, understanding the diverse symptoms of dementia is not straightforward, and family members and others involved in care frequently experience confusion and caregiver burden [5, 6, 7]. Because dementia is both a common condition and one that requires specialized care, a wide range of healthcare and long‐term care resources are necessary, including professionals with specific expertise in dementia care. The global economic burden of dementia, including direct medical and long‐term care costs as well as informal care costs such as lost work productivity among family caregivers, was estimated at USD 1.3 trillion in 2019 and is projected to increase to USD 2.8 trillion by 2030 [1].

Since the 1980s, research into the mechanisms and treatment of dementia has advanced rapidly. For Alzheimer's disease, which accounts for the largest proportion of dementia cases, substantial progress has been made in understanding molecular mechanisms, developing symptomatic treatments, implementing nonpharmacological interventions, promoting risk reduction strategies, and developing disease‐modifying therapies [8]. Alongside these advances, nuclear medicine imaging techniques that visualize brain pathology and blood‐based biomarkers reflecting pathological changes have increasingly been implemented in clinical practice [9]. More recently, anti‐amyloid monoclonal antibody therapies as disease‐modifying treatments have become commercially available and are now being used in clinical settings [10, 11]. Diagnostic methods and pathophysiological understanding have also advanced for other neurodegenerative diseases.

At the same time, improvements in diagnostic methods and an increased emphasis on early intervention have led to more frequent early diagnoses of dementia. Consequently, there is a growing need for long‐term support and care strategies that address daily life from the early stages of the disease onward.

Although pharmacological treatments continue to advance, current therapies cannot cure dementia, and nonpharmacological approaches remain essential for addressing the wide range of symptoms affecting daily life and psychological well‐being. Nonpharmacological approaches can be broadly categorized into three types. The first includes therapeutic interventions such as cognitive stimulation therapy, music therapy, exercise therapy, horticultural therapy, and reminiscence therapy [12]. The second focuses on care philosophies, including person‐centered care and validation approaches [13, 14, 15]. The third involves long‐term care services that support daily living through environmental modifications and continuous support. In addition to supporting people with dementia themselves, support for family members is also crucial, as dementia has often been described as a condition that affects not only the individual but also those close to them [16].

Beyond direct support for people with dementia and their families, stigma and prejudice toward dementia contribute to social exclusion and increased burden. Therefore, fostering societal understanding of dementia through education and awareness‐raising activities is also essential [17].

## 2 National Strategies and International Initiatives

Because dementia affects not only individuals but also families and society as a whole, and because research and development related to disease mechanisms, treatments, and care approaches are critically important, many countries have developed national dementia strategies and implemented comprehensive policy responses [18]. Countries such as France, the United Kingdom, Denmark, and the Netherlands introduced national dementia plans relatively early [19, 20]. In 2012, the World Health Organization identified dementia as a public health priority, and in 2017 published the “Global Action Plan on the Public Health Response to Dementia 2017–2025” [21, 22]. This action plan articulated a vision of a world in which all people with dementia can live with dignity, participate in society, and receive high‐quality care.

Japan is one of the most rapidly aging countries in the world, making the advancement of dementia policy an urgent issue. In this context, the following sections focus primarily on Japan's national initiatives while situating them within the broader international framework.

## 3 Evolution of Dementia Care Initiatives in Japan

In Japan, nationwide peer support organizations for families of people with dementia were established as early as 1980, even before the introduction of the Long‐Term Care Insurance (LTCI) system. At the same time, various initiatives were introduced to support the abilities of people with dementia in daily life, including the establishment of group homes in many regions. In 2000, the Long‐Term Care Insurance Act was enacted [23, 24, 25].

Japan's LTCI system assesses both physical and cognitive or mental care needs, enabling people with dementia to receive support from an early stage [26]. Taking into account the trend toward nuclear families, the system was designed not as a form of social welfare but as an insurance‐based system that facilitates access to care as a social right, thereby socializing caregiving responsibilities. Simultaneously, the adult guardianship system was implemented, establishing an institutional framework for rights protection and advocacy centered on people with dementia.

In 2005, revisions to the LTCI Act emphasized the concept of community‐based integrated care, promoting the provision of medical care, long‐term care, and daily living support within local living areas [27]. As a core component of this approach, Community General Support Centers were established in each municipality to provide early consultation, diagnostic support, care prevention, care planning for individuals requiring low levels of support, and responses to elder abuse [28]. During the same period, training programs to enhance dementia care competencies among primary care physicians and dementia supporter training programs for the general public were introduced. As of June 2025, more than 15 million people, over 12% of the total population, have completed dementia supporter training in Japan.

As a dementia‐specific national strategy, the Orange Plan was formulated in 2012 under the leadership of the Ministry of Health, Labour and Welfare, and was revised in 2015 as the New Orange Plan, a cross‐ministerial strategy [29]. The strategy was further updated over time, culminating in the proposal of the Dementia Policy Promotion Outline in 2019. Building on these cumulative efforts, the Basic Act on Dementia to Promote an Inclusive Society was enacted in June 2023 [30].

## 4 Specific Initiatives From the Orange Plan to the Basic Act on Dementia

The Orange Plan and New Orange Plan represented comprehensive national strategies that extended beyond the framework of the LTCI system. The original Orange Plan identified seven pillars: (1) development and dissemination of standardized dementia care pathways; (2) early diagnosis and early response; (3) development of medical services to support community living; (4) development of long‐term care services to support community living; (5) enhancement of daily life and family support in the community; (6) strengthening measures for young‐onset dementia; and (7) development of human resources engaged in medical and long‐term care services. These core components were largely retained in the New Orange Plan, with implementation carried out by municipalities and other stakeholders [29].

**FIGURE 1:**
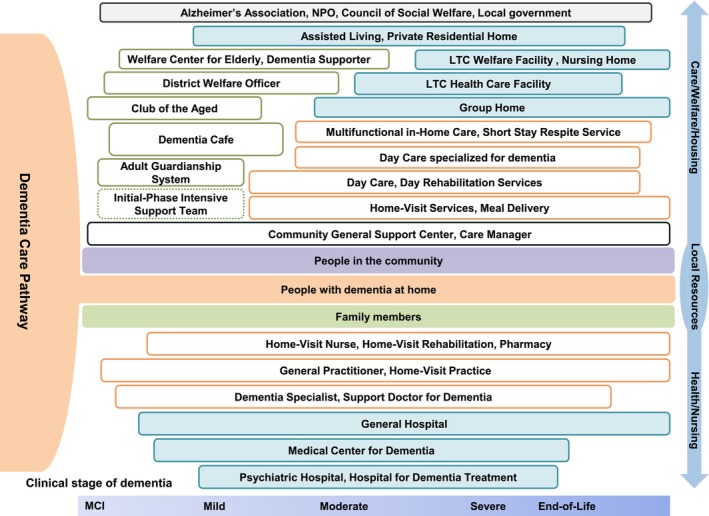
Conceptual framework of community‐based dementia care across the disease trajectory in Japan. This figure presents a conceptual framework illustrating how community‐based dementia care resources are organized and mobilized across the dementia trajectory, from early awareness to end‐of‐life care. At the center of the framework are people living with dementia and their families, emphasizing a person‐centered approach. The horizontal axis represents the progression of dementia over time, while the vertical structure distinguishes medical support from social, long‐term care, and community‐based support. Resources located closer to the center represent services that support continuity of daily life in community and home‐based settings, whereas resources positioned further outward indicate services activated with increasing care dependency, such as hospitalization or institutional care. The upper layer reflects social care, welfare, and community resources, many of which are delivered through the long‐term care insurance system, while the lower layer represents medical and diagnostic resources responding to disease progression. This framework highlights the dynamic interaction between medical and social care systems and illustrates how municipalities in Japan organize integrated dementia care by aligning available resources with changing needs over time. LTC, long‐term care; MCI, mild cognitive impairment; NPO, nonprofit organization.

As part of these initiatives, dementia care pathways were developed and disseminated as guidebooks that clearly present regional medical, long‐term care, and community resources from the early stages of dementia through end‐of‐life care. These guidebooks are distributed at municipal offices and Community General Support Centers and are used when residents seek consultation. Because the clinical course of dementia typically spans 5–15 years, dementia care pathways incorporate the concept of clinical pathways while encompassing a wide range of community resources (Figure 1). Although the general concept is shared internationally, the structure of dementia care pathways varies across countries depending on existing social resources and healthcare systems [31].

**FIGURE 2:**
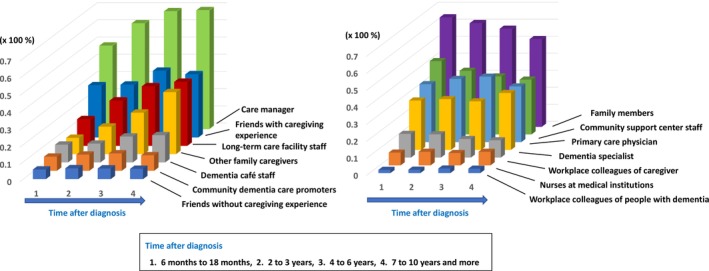
Consultation partners for family members of people living with dementia at and after diagnosis. This figure shows the results of an analysis of responses to the question, “Whom did you consult about dementia‐related issues or daily life after the diagnosis of dementia?” from the Survey on the experiences and support received by family members of people living with dementia conducted in 2021 (*n* = 2295). The post‐diagnostic period was divided into four phases: 6 months to 1.5 years, 2–3 years, 4–6 years, and 7 to ≥ 10 years after diagnosis. Consultation partners were categorized into 14 professional or social roles. The left side of the graph shows roles that tended to increase over time after diagnosis, whereas the right side shows roles that remained unchanged or decreased over time. The graph was created using data reported in Takechi et al. [32].

A nationwide survey conducted in 2022 examined how regional dementia‐related resources function as consultation points for families of people with dementia (Figure 2). Immediately after diagnosis, family members and Community General Support Centers were the most common sources of consultation. Over time, consultations increasingly involved care managers and staff at long‐term care service providers. Consultations at family caregiver support groups and dementia cafés also increased. Physicians, both primary care physicians and dementia specialists, played a consistent consultation role from diagnosis through end‐of‐life care [32]. These findings demonstrate that the multilayered roles of regional resources depicted in dementia care pathways are closely linked to actual support practices.

Following the New Orange Plan in 2015, dementia care teams were established in hospitals nationwide to strengthen care for hospitalized patients with dementia or delirium and to prevent hospital‐associated functional decline [33]. Additionally, early‐stage intensive support teams were established in municipalities across Japan to provide outreach services to people living in the community who have difficulty accessing dementia‐related medical and care services [34]. Dementia cafés were also established nationwide as accessible spaces where people with dementia and their families can seek advice and where continuous community‐based education about dementia is provided [35, 36, 37].

Building on the development of these initiatives, opportunities for people with dementia to share their experiences and perspectives have increased. Individuals living with dementia now serve as dementia ambassadors, delivering lectures and participating in meetings that convey the voices of those directly affected. The Japan Dementia Working Group was established to promote mutual support among people with dementia and to raise public awareness [38]. Initiatives such as “Orange Door,” in which people with dementia provide consultation to newly diagnosed individuals, have also emerged. Alongside these developments, the concept of dementia‐friendly communities, which has been promoted internationally, has increasingly taken root in Japan [39, 40, 41, 42].

## 5 Enactment of the Basic Act on Dementia

In June 2023, the Basic Act on Dementia to Promote an Inclusive Society was enacted, reflecting the accumulation of dementia policies and related practices [30, 43]. Article 1 of the Act states its purpose as promoting the realization of an inclusive and vibrant society in which all citizens, including people with dementia, can fully demonstrate their individuality and abilities while respecting and supporting one another. Article 3 outlines seven fundamental principles, including the enjoyment of fundamental human rights by people with dementia, the promotion of accurate public understanding of dementia, and appropriate support for families of people with dementia. Articles 4 through 8 define the responsibilities of the national government, local governments, medical and long‐term care providers, businesses related to daily living, and citizens in contributing to these principles.

Based on this Act, the Basic Plan for the Promotion of Dementia Policy was approved by the Cabinet in December 2024. The plan identifies four priority goals: (1) ensuring that all citizens understand a renewed perspective on dementia; (2) respecting the intentions and preferences of people with dementia in their daily lives; (3) enabling people with dementia and their families to live safely in the community while supporting one another; and (4) enabling citizens to utilize new knowledge and technologies related to dementia. The plan also emphasizes the importance of establishing key performance indicators to evaluate policy effectiveness and revising measures based on these evaluations.

Following the enactment of the Basic Act and the formulation of the Basic Plan, the “Guidelines for Decision‐Making Support in Daily and Social Life for People with Dementia”, originally published in 2018, were revised and released as a second edition in March 2025 [44, 45]. The revised guidelines strongly reflect the renewed perspective on dementia, emphasizing that people with dementia continue to have abilities and aspirations. They place particular emphasis on human rights and social inclusion. The guidelines highlight the importance of creating environments that support the expression of decision‐making preferences and of providing support from the perspective of the individual rather than the supporter. They also emphasize supported decision‐making rather than proxy decision‐making, which has recently gained increasing emphasis at the international level [46].

Japan's enactment of the Basic Act on Dementia and related guideline revisions represent a proactive advancement of the principles outlined in the WHO Global Action Plan on the Public Health Response to Dementia 2017–2025.

## 6 Challenges and Future Directions

This review has outlined the evolution of dementia policies and care practices globally and in Japan. While progress has been made, the proportion of countries that have established national dementia strategies remains approximately 30.8%, far short of the WHO target of 75%. Continued international collaboration and information sharing are needed to expand access to standardized dementia care worldwide [1].

### 6.1 Measuring Outcomes From the Perspective of People With Dementia

In Japan, initiatives linked to the LTCI system, adult guardianship system, and dementia policies since the Orange Plan have contributed to environments that respect the dignity of people with dementia. Nevertheless, significant challenges remain. These challenges stem not only from incomplete policy implementation but also from the inherent complexity of dementia itself. For example, evaluating outcomes from the perspective of people with dementia is difficult, particularly for those in advanced stages who may have limited ability to express their views. Even individuals in the mild to moderate stages may find it challenging to articulate their experiences in light of objective circumstances. There are likely many aspects of this issue to which the concept of supported decision‐making can be applied [46].

### 6.2 To Enhance Community Residents' Understanding of Dementia

Public education and awareness activities continue, but understanding the diverse symptoms of dementia in a simplified manner remains difficult, as does measuring and improving public understanding. To address this, a self‐assessment tool has been proposed to quantitatively capture key competencies involved in interacting with people with dementia [47]. Such indicators may help evaluate and enhance community‐based initiatives.

Although dementia supporter training is widespread in Japan, further emphasis on translating knowledge into concrete actions may be beneficial. In the United Kingdom, the Dementia Friends program requires participants not only to complete training but also to commit to specific actions [48]. Incorporating similar elements into Japan's approach may enhance impact.

### 6.3 Caregiver Burden and Elder Abuse: Toward Realizing “No One Left Behind”

Additional challenges include monitoring and reducing caregiver burden and preventing employment loss due to caregiving responsibilities. Reports indicate that cases of elderly abuse continue to increase annually, with dementia being a major contributing factor [49, 50]. Reducing elderly abuse should therefore be considered a key indicator of progress toward an inclusive society in which no one is left behind.

### 6.4 Reducing Local Disparities in Dementia Policy Implementation

There is also room for improvement in the implementation of dementia policies. Although many initiatives are undertaken at the municipal level, substantial variation exists in the scope and quality of activities. For example, early‐stage intensive support teams vary widely across municipalities. Centralized training and coordination systems, such as the Admiral Nurse model in the United Kingdom, may offer a pathway toward greater standardization [51, 52].

### 6.5 Balancing Costs, Quality of Life, and Dignity in Dementia Care

As aging progresses and the number of people with dementia increases, the costs associated with dementia care are expected to rise. From the perspective of social security sustainability, careful discussion of appropriate strategies is essential. The long‐term impact of newly introduced disease‐modifying therapies for Alzheimer's disease, whether they will enhance quality of life and dignity for people with dementia and their families or primarily increase healthcare costs, must be monitored over time.

## 7 Conclusion

Dementia care has evolved from isolated initiatives to comprehensive national strategies, leading to significant changes in policy and practice. Public perceptions of dementia have also shifted, contributing to improved quality of life and dignity for people with dementia and their families. Nevertheless, substantial challenges remain, and continued efforts are required to further advance dementia care in aging societies. Continued international dialogue and empirical evaluation will be essential to translate policy frameworks into meaningful improvements in the daily lives of people living with dementia.

## Disclosure

The author has nothing to report.

## Ethics Statement

Ethics approval was not applicable for this review.

## Data Availability

Data sharing is not applicable to this article as no datasets were generated or analyzed during the current study.
